# S52. WORKING MECHANISMS OF VIRTUAL REALITY BASED CBT FOR PARANOIA: A RANDOMIZED CONTROLLED TRIAL EXAMINING COGNITIVE BIASES, SCHEMATIC BELIEFS AND SAFETY BEHAVIOR

**DOI:** 10.1093/schbul/sby018.839

**Published:** 2018-04-01

**Authors:** Chris Geraets, Marije Van Beilen, Roos Pot-Kolder, Mark Van der Gaag, Wim Veling

**Affiliations:** 1 University Medical Center Groningen; 2 VU Amsterdam

## Abstract

**Background:**

Recently, the efficacy of a novel virtual reality based cognitive behavior therapy (VR-CBT) for paranoia was demonstrated. Cognitive biases, cognitive limitations, negative schematic beliefs and safety behavior have been associated with paranoid ideations and delusions. It is unknown whether VR-CBT affects these associated factors, and how changes in these factors relate to changes in paranoid ideation.

**Methods:**

In this multi-center randomized controlled trial patients with a psychotic disorder and paranoia were randomized to VR-CBT (n = 58) or treatment as usual (TAU; n = 58). VR-CBT consisted of maximally sixteen 60-minute individual therapy sessions. Paranoia, safety behavior, schematic beliefs, cognitive biases and limitations were assessed at baseline, post-treatment (at three months) and follow-up (at six months). Mixed model analyses were conducted to study treatment effects. Mediation analyses were performed to explore putative working mechanisms by which VR-CBT reduced paranoia.

**Results:**

VR-CBT, but not TAU, led to reductions in jumping to conclusions, attention for threat bias and social cognition problems. Schematic beliefs remained unaffected. The effect of VR-CBT on paranoia was mediated by reductions in safety behavior and social cognition problems.

**Discussion:**

VR-CBT affects multiple mechanisms that are associated with paranoid ideation. Although maintaining factors of paranoia are likely to influence each other, targeting safety behavior and social cognitive problems seems effective in breaking the vicious circle of paranoia.

